# Catastrophic Health Expenditure and Mental Health in the Older Chinese Population: The Moderating Role of Social Health Insurance

**DOI:** 10.1093/geronb/gbab130

**Published:** 2021-07-13

**Authors:** Wei Yang, Bo Hu

**Affiliations:** 1 Department of Global Health and Social Medicine, King’s College London, UK; 2 Care Policy and Evaluation Centre (CPEC), Department of Health Policy, The London School of Economics and Political Science, UK

**Keywords:** Catastrophic health expenditure, China, Mental health, Older people, Social health insurance

## Abstract

**Objectives:**

Catastrophic health expenditure (CHE) has considerable effects on household living standards, but little is known regarding the relationships between CHE and people’s mental health. Using China as an example, this study examines the association between CHE and mental health and investigates whether the association differs between those with and without social health insurance (SHI).

**Methods:**

The data came from 3 waves of the China Health and Retirement Longitudinal Study (2011, 2013, and 2015, *N* = 13,166). We focused on older people aged 60 and older. We built panel data regression and quantile regression models to analyze the data.

**Results:**

Incurring CHE is significantly associated with poor mental health. The association is weakened among older people receiving SHI, which indicates that SHI has a protective effect. Moreover, the association between CHE and mental health and the protective effect of SHI are stronger among those with mild or moderate mental health problems.

**Discussion:**

Our findings provide empirical evidence that encourages the integration of psychologically informed approaches in health services. We also urge governments in low- and middle-income countries to consider more generous health financing mechanisms for older people with greater health care needs.

Governments around the world have been concerned with protecting people from catastrophic health expenditure (CHE)—defined as health spending that exceeds a predefined percentage of households’ ability to pay for health care. Although out-of-pocket (OOP) payments for health have been falling, OOP payments as a share of income have not been declining. It is estimated that, globally, 808 million people incurred CHE in 2015 (Wagstaff et al., 2018). In low- and middle-income countries (LMICs), even a small amount of health expenditure can lead to undue financial hardship for poor households. These households often have to borrow money or reduce other basic household expenditure on items such as food or accommodation to meet this health expenditure.

Existing studies show that CHE has considerable effects on household living standards (Wagstaff et al., 2003; Xu et al., 2003; Yang & Wu, 2014), but little is known about the relationship between CHE and people’s mental health. The existing evidence suggests that there is a clear association between socioeconomic status (SES) and mental well-being, and the prevalence of mental health problems is higher among poorer people (Alegria et al., 2018). However, previous studies do not take into account changes in financial status caused by illness. As paying for treatments may lead to a drastic deterioration in a household’s financial status, individuals may feel that they are threatened by the prospect of severe financial losses. Existing data show that financial worries are among the most troubling for households, and these worries have been suggested to be robustly linked to a range of mental health outcomes, such as psychological distress, anxiety, and depression (Fiori et al., 2016; Rohde et al., 2016; Sjoberg, 2010, 2014).

Social health insurance (SHI) is a state-centered solution to the problem of incurring a large amount of OOP health expenditure. Originating in Europe, mature SHI systems often require working-age adults and their employers as well as the self-employed to make contributions that will cover a package of health services that are available to the insured participants and their dependents. One of the most important functions of SHI is to protect health care recipients suffering from financial catastrophe and improve access to care.

In this article, we examine a crucial but often overlooked function of SHI: By reducing the economic burden of health care and providing economic certainty in the future, SHI may have a positive influence on people’s mental health. First, SHI can protect households from incurring direct vital financial losses. Households incurring direct financial losses may adjust their standard of living or even spend their savings. These consequences will trigger various types of mental health problems, such as anxiety and depression. By lessening the degree of losses, SHI can enhance an individual’s ability to plan and control his or her life. Second, SHI may reduce people’s sense of financial vulnerability to potential illness. People who have incurred CHE often have a higher probability of falling ill again in the near future. Enrollment in an SHI scheme protects against financial losses and reduces uncertainty in regard to financial outcomes in the future, which improves mental health by giving people a sense of security.

We focus our study on older people for the following reasons. First, as people age, their health may deteriorate more quickly compared to younger adults, and many older people suffer from longstanding illnesses or chronic conditions that require regular health care. Therefore, older people can be intensive users of health care services and often have a higher risk of incurring large health care costs (Yang, 2020). Second, older people are more vulnerable to a financial shock, such as CHE, due to a loss of productivity or shrinking of income. As a result, they are often more likely to incur CHE (Esping-Andersen, 2002). This study uses China as a case study, as China has an established SHI system that has covered most of its population since the 2000s. Ample evidence suggests that China’s SHI has reduced CHE and improved financial security (Wang et al., 2020; Zhang et al., 2019). However, very little is known about the relationship between CHE and mental health among older people in this country, and even less is known about the impacts of SHI on mental health. Drawing on data from the China Health and Retirement Longitudinal Study (CHARLS), this study extends previous work by examining the relationship between CHE and mental health and the moderating roles of SHI in the older Chinese population.

## Literature Review

### Mental Health, CHE, and SHI

Mental health is crucial for people to live a fulfilling life. Previous studies have identified physical health and social support as the key predictors of mental health. On the one hand, the onset of illness and a decline in functional capability cause major stress in a person’s life and result in heightened risks of mental disorders (Williamson, 1998). On the other hand, social support networks are valuable resources that people rely upon to cope with a stressful situation. The quality and size of support networks have a positive influence on multiple dimensions of mental health, such as life satisfaction, quality of life, and happiness (Nyqvist et al., 2013), whereas a lack of support can result in severe mental health problems (Hu & Wang, 2019).

Mental health is also affected by financial resources and status. Economic hardship imposes a great strain on people’s mental well-being because it renders even the most basic needs, such as food and housing, unfulfilled. In addition, financial resources indicate a person’s capability to fend off the negative impacts of other stressors (e.g., poor physical health) on mental health. Existing evidence provides robust support for the negative impacts on mental health of exposure to economic risks. It has been reported that people’s level of income and wealth are positively correlated with their mental health (Hu, 2021; Kourouklis et al., 2019; Wilkinson, 2016). Rohde et al. (2016) examined a series of indicators of financial insecurity, such as financial dissatisfaction, reductions and volatility in income, and an inability to meet standard expenditure, and found that they are likely to be hazardous to mental health. Similar findings were reported in Hiilamo and Grundy’s (2020) work. They found that mental health status is greatly worsened among people with substantial financial debt.

SHI offers household protection against future health risks. Unlike private health insurance, which is often voluntary and has restrictions on enrollment and benefits depending on the insurance program, SHI is usually compulsory, centrally administered by the government, and can be provided against risks that private insurance does not cover. Numerous studies have demonstrated the positive effects of SHI on the reduction of OOP health payments and consequently CHE and health payment-induced poverty, but the mechanism by which SHI relates to mental health status has not been specified in the literature. The existing evidence points to relevant perspectives in understanding this relationship. As a loss of income, or a change in financial status, and the associated negative events may lead people to feel that they have a lack of control over their own life, studies have demonstrated that social/welfare policies can improve mental health status through the increased availability of financial resources to cope with adverse economic and social conditions. For instance, Sjoberg (2017) and Di Tella et al. (2003) examined sickness benefits that provided financial support during times of illness and concluded that generous sickness benefits had long-term impacts on self-reported life satisfaction and individual health trajectories and helped people to recuperate from illness.

Past CHE experience may be associated with future mental health status by influencing perceptions of future CHE risk, and policies aimed at reducing potential financial losses may help to improve financial security and consequently mental health. Using a social experimental design, Weathers and Stegman (2012) found that expanding the coverage of a newly entitled Social Security Disability Insurance in the United States led to substantial improvements in mental health in the short run. They also suggested that short-term improvements in mental health may lead to longer-term reductions in other health impairments and mortality. Sjoberg (2010, 2014) focused on old-age pensions and public unemployment insurance and found that these policies may lessen the negative effects of financial insecurity on individuals’ subjective well-being.

### The Case Study of China

We chose China as a case study country for this article. China is a rapidly aging country. The total number of those aged 65 and older stood at approximately 110 million, or 8% of the entire population, in 2010. The proportion of older people is projected to reach 26% by 2050, exceeding that of most European Union countries (Hu, 2019). Although the proportion of health expenditure accounting for total household income/expenditure has been decreasing over the years (Yang, 2020), evidence has shown that a significant proportion of the population in China still suffer from CHE, especially those without SHI or those who have a chronic illness (Goeppel et al., 2016; Gwatidzo & Stewart Williams, 2017).

Older people living with mental health problems in China, a demographic group comprising more than 20% of the country’s population, have been neglected until recently (Que et al., 2019). Studies have shown that the proportion of older people suffering from mental health problems is increasing. In 2011, approximately 30% of men and 43% of women aged 45 and older in China had some depressive symptoms. The percentage is even higher for those aged 60 and older, and it exceeds some other Asian countries, such as Indonesia (Lei et al., 2014). The findings regarding the relationship between the financial status and mental health of older people in China are consistent with the international literature. Hsieh (2015) found that older people with a higher financial status were less likely to have depression. Liu et al. (1998) suggested that economic stability and health care investments had positive effects on both the physical and mental health of the population. Zhang and Gao’s study (2021) found that for those who had an incidence of CHE, mental health status decreased by 0.10 and 0.30 units for older people living in rural and urban areas, respectively, and functional limitations increased by 0.14 units for older people living in urban areas.

One important objective of this study was to explore whether the mental health conditions of older people with SHI are different from those without insurance in the presence of CHE. Next, we provide some background information on China’s SHI system for the discussion. China currently has an established SHI system that comprises three different SHI schemes: (a) the Urban Employee Insurance (UEI), covering urban residents with formal employment, (b) the Urban Resident Insurance (URI), covering urban residents without formal employment, and (c) the New Rural Cooperative Medical Scheme (NCMS), covering rural residents. Among the three SHI programs, the UEI has the most generous benefit packages and offers the highest reimbursement rates, as UEI participants are required to pay monthly premiums through their payroll into the insurance program, and employers are also required to contribute. The URI and the NCMS have the least comprehensive benefit packages as participants of these two insurance programs are either without formal employment or work in the informal sector, and the URI and the NCMS are heavily subsidized by the local or central governments (Tan et al., 2019). In recent decades, the Chinese government has started to consolidate the URI and the NCMS into a more comprehensive SHI scheme—the Urban and Rural Health Insurance—but the progress of the consolidation has been uneven across the country.

Ample evidence has demonstrated positive effects of SHI on physical health and a reduction of health payments (Abel-Smith, 2014; Meng et al., 2015; Wang et al., 2020), but evidence of the effect of SHI on mental health is rather scant. We found only one article looking at the effect of SHI on people’s mental health. Li and Yuan (2019) examined the change in mental health status when older people with chronic diseases transited from a more generous SHI to a less generous one. The study found that the experience of transition leads to significant deterioration in mental health, and one possible explanation is that transition conflicts with continuous treatment of chronic disease conditions, which can result in the worsening of mental health for chronic disease patients. So far, no studies have yet examined the role of SHI in moderating the relationship between significant financial losses caused by CHE and mental health. Furthermore, very little is known about such a relationship among older people in China. Based on the discussion above, this study seeks to answer the following two research questions:

1. Is CHE associated with poor mental health among older people in China?
2. Does SHI have a positive influence on older people’s mental health in the presence of CHE?

## Method

### Data

This study uses data from the three waves of the CHARLS. Following a four-stage cluster sampling procedure, the CHARLS is a nationally representative survey that collects health and aging-related information from Chinese people aged older than 45. The baseline survey took place in 2011, with two follow-up surveys conducted in 2013 and 2015. In addition to interviewing the same people who participated in the baseline survey, a refreshed sample was interviewed to maintain the representativeness of the sample in the follow-up surveys. Our analysis focused on 13,166 older people older than 60 years of age who had used any health services in the pooled sample.

### Key Measurements

Mental health, the outcome variable in our analysis, is measured by people’s depressive symptoms. The CHARLS questionnaire contains the 10-item Center for Epidemiologic Studies—Depression (CES-D) scale. Survey participants were asked to rate eight negative statements (e.g., I felt fearful) and two positive statements (e.g., I felt hopeful), which indicated their mental health status for the past week. Each statement was measured on a 4-point scale: 1 (*less than one day*), 2 (*one to two days*), 3 (*three to four days*), and 4 (*five to seven days*). We reverse-scored the two positive statements and added up the scores of the 10 statements, which enabled us to create a depressive symptom variable ranging from 10 (*no symptoms*) to 40 (*severe symptoms*).

The key independent variables of interest are CHE and SHI. We included two measures of CHE. We first calculated the proportion of OOP health expenditure a person spent on health care in the previous year out of the annual household expenditure per capita. We used 10%, 20%, and 25%, respectively, as the threshold to determine a case of CHE (Wagstaff et al., 2018; Xu et al., 2003). The OOP health expenditure was adjusted for inflation and expressed in 2011 prices using the Consumer Price Index (Chinese National Bureau of Statistics, 2020). A dichotomous variable of CHE incidence was constructed if the proportion of health expenditure exceeded the defined thresholds. Second, we examined whether spending a higher proportion of household expenditure on health care is associated with a worsened mental health status. In this case, we used the actual proportion of OOP health expenditure as the independent variable in our regression model. The CHARLS questionnaire asked respondents whether they were receiving any SHI, including UEI, URI, NCMS, government medical insurance, or urban and rural combined insurance. We created a dichotomized variable: 0 = no SHI and 1 = receiving SHI.

### Control Variables

We controlled for the variables that might confound the relationships between mental health and the key independent variables of interest (i.e., catastrophic health care expenditure and receiving medical insurance). The selection of the control variables was based on the existing literature subject to data availability. We examined people’s physical health, sociodemographic characteristics, SES, and lifestyle factors. Regarding physical health, we controlled for functional capability, often feeling pain, number of chronic diseases, and self-reported health. The CHARLS questionnaire asked survey participants whether they could perform six activities of daily living (ADLs; eating, dressing, bathing, using the toilet, continence, and getting in and out of bed) and six instrumental activities of daily living (IADLs; cooking, shopping, making phone calls, taking medication, managing money, and doing housework). We included continence as a limitation for ADL because the ability to control one’s bladder has been used in the literature as a basic item of ADL, and this item is associated with multiple chronic conditions that may lead to disabilities or functional limitations, such as stroke and Alzheimer’s disease (Edemekong et al., 2020; Katz et al., 1970). Each item was measured on a 4-point scale: 1 (*I do not have difficulty*), 2 (*I have difficulty but can do it*), 3 (*I need help*), and 4 (*I cannot do it*). We considered people who needed help with a particular task or could not do it themselves as having a functional limitation in relation to this task. We added up the number of ADL limitations and IADL limitations reported by survey participants.

A frequent feeling of pain is a dichotomized variable: 0 = no and 1 = yes. The self-report health variable has three categories: very good health, good health, and fair or poor health. The CHARLS survey asked participants whether they had any of the chronic diseases such as hypertension or diabetes. We created a variable by adding up the number of chronic diseases each person reported in the survey.

We controlled for three sociodemographic factors: age, gender, and marital status. The marital status variable has two categories: 0 = separated, widowed, divorced, or never married and 1 = married or cohabiting with a partner. The SES variables examined in the analyses were education, household expenditure per capita in 2011 prices, and total health expenditure in 2011 prices. The education variable has three categories: no formal education, primary or secondary education, and high school education or above. Total health expenditure included OOP expenditure and expenditure paid through SHI. We controlled for two lifestyle variables: smoking (1 = current smoker and 0 = nonsmoker) and drinking (1 = more than once per month and 0 = once per month or none) in the regression. We followed Gong et al.’s (2016) work to construct the drinking variable.

### Statistical Analysis

Taking advantage of the longitudinal feature of the CHARLS data, our base case analysis, which aimed to answer the first research question (RQ 1), was a panel data model specified as follows (Equation 1):


$$\begin{array}{l} y_{it}\;=\;\beta_{0}+\;\beta_{1}\times c_{it}+\beta_{2}\times m_{it}+\underset{k=3}{\overset{K}{\sum }}\;(\beta_{k}\times z_{it}^{k})+\Delta_{i}+\epsilon_{it}\;\;\; \end{array}$$ (1)

where $y_{it}$ denotes the outcome variable, which is the depressive symptoms score for an individual *i* in wave *t* (*t* = 1–3). $c_{it}$ denotes the incidence of CHE or the intensity of OOP expenditure, and $m_{it}$ denotes whether or not SHI is received. $z_{it}^{k}$ denotes the control variables. $\beta_{k}$ are the coefficients of the respective regressors to be estimated from the model. $\Delta_{i}$ captures the unobserved individual-level heterogeneity, and $\epsilon_{it}$ is the error term.

We investigated both the random-effects (RE) and fixed-effects (FE) panel data models. A RE model assumes that $\Delta_{i}$ is not correlated with any of the regressors and thus may be vulnerable to estimation bias. A FE model does not rely on such an assumption, but FE estimators can be imprecise (i.e., large standard errors), especially when intraindividual variations are low in comparison to interindividual variations (Cameron & Trivedi, 2005, p. 715). We calculated the cluster-robust standard errors to account for the intraindividual correlation in the sample.

To answer the second research question (RQ 2), we added the interaction term between CHE and SHI to Equation 1 and examined the role of SHI in the relationship between CHE and depressive symptoms (Equation 2).


$$\begin{array}{ll} y_{it}\;= & \;\beta_{0}+\beta_{1}\times c_{it}+\beta_{2}\times m_{it}+\beta_{3}\times c_{it}\times m_{it}\\ & +\underset{k=4}{\overset{K}{\sum }}\;(\beta_{k}\times z_{it}^{k})+\Delta_{i}+\epsilon_{it}\;\; \end{array}$$ (2)

To further investigate the heterogeneity in the relationship between CHE, SHI, and depressive symptoms, we employed quantile regression analyses. The θ-th conditional quantile function of the outcome variable is specified as follows (Equation 3):


$$\begin{array}{ll} Q_{\theta }\left(y_{it}\right)\;= & \;\beta_{0}\left(\theta \right)+\beta_{1}\left(\theta \right)\times c_{it}+\beta_{2}\left(\theta \right)\times m_{it}\\ & +\beta_{3}\left(\theta \right)\times c_{it}\times m_{it}+\underset{k=4}{\overset{K}{\sum }}\;(\beta_{k}(\theta )\times z_{it}^{k})\;\;\; \end{array}$$ (3)

$\beta_{k}(\theta )$ denotes the coefficients in relation to the θ-th quantile function. In this study, we report the regression results for the 10th, 30th, 50th (median), 70th, and 90th quantiles. The denotations of $c_{it}$, $\;m_{it}$, and $z_{it}^{k}$ are the same as in Equation 1. We calculated the heteroskedasticity-robust standard errors.

We conducted supplementary analyses to check the robustness of our results. First, we built lagged regression models where CES-D scores in Waves 2 and 3 (i.e., CHARLS 2013 and 2015) were outcome variables and CES-D scores in the baseline survey (i.e., CHARLS 2011) were included as another control variable. This was to rule out the possibility that the association between CHE and mental health in the base case analysis (RQ 1) was purely due to the initial status of mental health. It should be noted that the lagged regression analysis was based on 4,404 out of 13,166 older people in our sample who participated in both the baseline and follow-up surveys. Second, we investigated the relationships between CHE incidence and mental health among older people receiving SHI and those not receiving it, respectively. We compared the coefficients of CHE incidence for these two groups of people to evaluate the role of SHI (RQ 2) from another angle.

## Results

**Figure 1.**
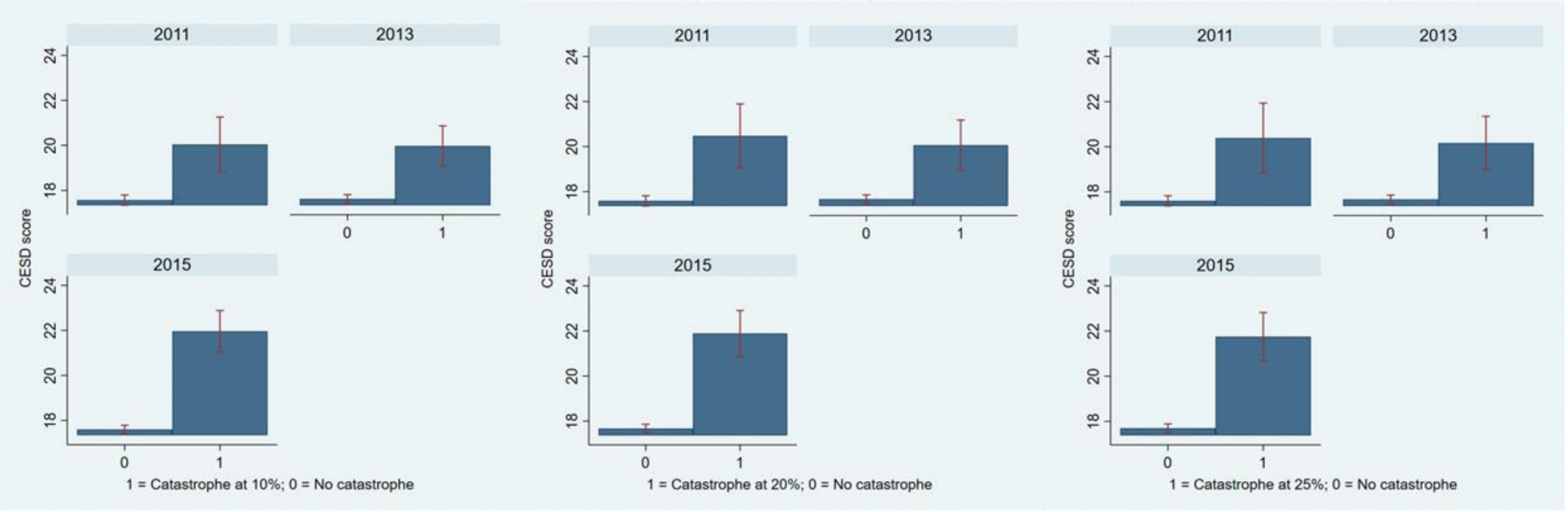
Mental health of older people according to different thresholds of catastrophic health expenditure incidence (mean and 95% confidence interval). CES-D = Center for Epidemiologic Studies—Depression.

Figure 1 compares the mean CES-D scores between those who incurred CHE and those who did not (with 95% confidence intervals plotted on the mean) at the threshold levels of 10%, 20%, and 25%, respectively. It can be noted that the CES-D scores were significantly higher for those who incurred CHE than those who did not, regardless of the threshold. In other words, those without CHE had better mental health compared to those who had CHE.

Table 1 compares the sample characteristics of people who incurred CHE at the threshold level of 10% and those who did not. Individual weights with nonrespondent adjustment were applied for the summary statistics. At the 10% threshold, there were 731 cases of CHE. As expected, people incurring CHE had much worse health than those who did not in terms of self-reported health, number of chronic diseases, functional capability, and frequent feeling of pain. The two groups of people are similar in terms of demographic characteristics. People incurring CHE on average spent 12,800 yuan ($1,900) on health care in the previous year. OOP expenditure on average amounted to 7,700 yuan ($1,200).

**Table 1.** Sample Characteristics

	Full sample	No CHE	CHE (10% threshold)
Variables	Mean or proportion (weighted)		
CES-D score	17.4	17.3	20.5
Insured by SHI (%)	94%	94%	95%
Age (years old)	66.9	66.8	67.7
Male (%)	46%	46%	46%
Rural China (%)	55%	55%	57%
Education level (%)			
No education	50%	50%	50%
Secondary education or below	40%	40%	41%
High school or above	10%	10%	10%
Married (%)	96%	96%	98%
Self-assessed health (%)			
Very good	14%	15%	3%
Good	32%	33%	13%
Fair or poor	54%	52%	84%
Number of chronic diseases	1.5	1.5	2.4
Number of ADL limitations	0.1	0.1	0.4
Number of IADL limitations	0.3	0.3	0.8
Having pain (%)	33%	32%	52%
Smoking (%)	24%	24%	15%
Drinking (%)	27%	28%	15%
Household expenditure per capita (thousand yuan)	10.7	10.7	11.0
Out-of-pocket health expenditure (thousand yuan)	0.40	0.03	7.66
Total health expenditure (thousand yuan)	0.79	0.11	12.8
Sample size	13,166	12,435	731

*Notes:* CHE = catastrophic health expenditure; CES-D = Center for Epidemiologic Studies—Depression; SHI = social health insurance; ADL = activities of daily living; IADL = instrumental activities of daily living. Total health expenditure includes out-of-pocket health expenditure and expenditure paid by the government.

Table 2 presents the effects of CHE incidence (i.e., incurring CHE or not) on the depression scores. At the 10% threshold, incurring CHE is associated with poorer mental health. The coefficient is statistically significant in both the RE and FE models, regardless of whether the interaction term is included in the models. The coefficient of the interaction term between CHE incidence and SHI is negative, indicating that the negative association between CHE incidence and mental health is attenuated among people receiving SHI. The coefficient is statistically significant in the RE model only (β = −2.40, *p* < .05), although the effect size is larger in the FE model (β = −2.65, *p* = .07). The association between CHE and poor mental health remains statistically significant when we use 20% or 25% as an alternative threshold of catastrophic expenditure. In addition, at the 20% threshold, the interaction term is statistically significant in both the RE (β = −2.21, *p* < .05) and FE models (β = −3.24, *p* < .05). The regression results of the full models with control variables are presented in Supplementary Appendix (Table A1).

**Table 2.** Association Between CHE Incidence, Mental Health, and SHI: Panel Data Regression and Quantile Regression

	Threshold of catastrophic health expenditure: 10%								
	Base case analysis		Moderation analysis		Quantile regression analysis				
	Random effects	Fixed effects	Random effects	Fixed effects	0.1	0.3	0.5	0.7	0.9
Incidence of CHE	1.40*** (0.31)	0.92* (0.37)	3.68*** (0.98)	3.43* (1.44)	4.45** (1.36)	2.83*** (0.84)	4.20* (1.94)	3.90* (1.69)	1.24 (1.28)
Having SHI or not	−0.24 (0.24)	−0.01 (0.37)	−0.14 (0.24)	0.12 (0.37)	0.11 (0.13)	−0.06 (0.25)	−0.17 (0.31)	−0.74* (0.34)	−1.17* (0.51)
SHI × Incidence of CHE			−2.40* (1.00)	−2.65 (1.46)	−3.93** (1.40)	−1.70 (0.87)	−2.78 (1.95)	−2.33 (1.71)	1.88 (1.30)
Controls	Yes	Yes	Yes	Yes	Yes	Yes	Yes	Yes	Yes
	Threshold of catastrophic health expenditure: 20%								
	Base case analysis		Moderation analysis		Quantile regression analysis				
	Random effects	Fixed effects	Random effects	Fixed effects	0.1	0.3	0.5	0.7	0.9
Incidence of CHE	1.50*** (0.37)	0.93* (0.43)	3.61*** (1.09)	4.01** (1.31)	5.17*** (0.99)	2.75 (2.07)	2.60 (3.83)	2.61 (2.12)	1.14 (0.58)
Having SHI or not	−0.24 (0.24)	−0.01 (0.37)	−0.17 (0.24)	0.09 (0.37)	0.17 (0.12)	−0.11 (0.25)	−0.22 (0.30)	−0.82* (0.34)	−1.15** (0.45)
SHI × Incidence of CHE			−2.21* (1.11)	−3.24* (1.35)	−4.04*** (1.14)	−1.61 (2.08)	−1.13 (3.83)	−1.08 (2.17)	2.21** (0.69)
Controls	Yes	Yes	Yes	Yes	Yes	Yes	Yes	Yes	Yes
	Threshold of catastrophic health expenditure: 25%								
	Base case analysis		Moderation analysis		Quantile regression analysis				
	Random effects	Fixed effects	Random effects	Fixed effects	0.1	0.3	0.5	0.7	0.9
Incidence of CHE	1.45*** (0.40)	0.96* (0.46)	2.74** (1.03)	2.95* (1.28)	5.15*** (1.20)	1.85 (1.75)	0.81 (2.20)	1.94** (0.64)	0.07 (2.16)
Having SHI or not	−0.24 (0.24)	−0.01 (0.37)	−0.20 (0.24)	0.04 (0.37)	0.16 (0.13)	−0.1 (0.25)	−0.21 (0.30)	−0.83* (0.33)	−1.25** (0.45)
SHI × Incidence of CHE			−1.36 (1.06)	−2.08 (1.32)	−4.01** (1.28)	−0.62 (1.77)	0.56 (2.21)	−0.52 (0.79)	3.22 (2.17)
Controls	Yes	Yes	Yes	Yes	Yes	Yes	Yes	Yes	Yes

*Notes:* CHE = catastrophic health expenditure; SHI = social health insurance. Cluster/heteroskedasticity-robust standard errors are presented in parentheses.

*p < .05, **p < .01, ***p < .001; *N* = 13,166.

We further explored the negative association between CHE incidence and mental health as well as the protective effects of SHI across different quantiles of the CES-D scores (Table 2). There are several important observations to note. At the 10% threshold, the association between CHE and SHI was significant at the lower quantiles, but not at the highest quantile. Similarly, the protective effect of SHI was significant for the lower quantiles (≤0.5), or in other words, people with better mental health. Specifically, at the 0.1 quantile, among those who were covered by SHI, the CES-D score for those incurring CHE was 0.52 units (= 4.45 − 3.93) higher than those who did not incur CHE; for the uninsured, the CES-D score for those incurring CHE was 4.45 units higher compared to those who did not incur CHE. A significant decrease in the negative association between CHE and SHI for the lower quantiles was also found when the CHE threshold was set at 20% or 25%.

Having identified the varying association between CHE incidence, SHI, and mental health, we focused on whether the intensity of OOP health expenditure has any effects on people’s mental health and whether the influence of SHI we found in the earlier analysis still exists. Table 3 presents the regression results of the two models that are based on people who used health services in the past 12 months. The coefficient of the intensity of OOP health expenditure is positive and statistically significant in both the RE (β = 0.41, *p* < .01) and FE models (β = 0.51, *p* < .001). This suggests that people incurring a higher proportion of OOP health expenditure have worse mental health, especially for those without SHI. The coefficient of the interaction term between the intensity of OOP health expenditure and receiving SHI is negative but not statistically significant. The results of the full models with control variables are presented in Supplementary Appendix (Table A2).

**Table 3.** Association Between Intensity of OOP Health Expenditure, SHI, and Mental Health

	Panel data regression	
	Random effects	Fixed effects
OOP health expenditure	0.41** (0.16)	0.51*** (0.15)
Having SHI or not	−0.18 (0.24)	0.04 (0.37)
SHI × OOP health expenditure	−0.19 (0.25)	−0.49 (0.31)
Controls	Yes	Yes

*Notes:* OOP = out-of-pocket; SHI = social health insurance. Clustered standard errors are presented in parentheses.

**p < .01, ***p < .001.

Table 4 reports the results of the robustness checks. The results in the upper panel show that people’s mental health status in the baseline survey is a significant predictor of that in later waves, which reflects strong intertemporal correlations of mental health. However, even after we controlled for the CES-D scores at the baseline, the coefficient of CHE incidence remained statistically significant, regardless of the threshold of CHE. It seems that we could rule out the possibility that the strong association between CHE incidence and CES-D scores observed in Table 2 is entirely attributable to baseline differences in mental health. The lower panel of Table 4 shows that, at the 10% threshold level, the coefficient of CHE incidence is 3.08 (*p* < .05) for people not receiving SHI, whereas that for people receiving SHI is 1.05 (*p* < .001). The former coefficient is twice as large as the latter. The Chow test shows that their difference is statistically significant (*p* < .05). Such a result is consistent with that given in Table 2: receiving SHI attenuates the association between CHE incidence and poor mental health.

**Table 4.** Robustness Checks

Controlling for baseline CES-D scores in lagged models	10% threshold	20% threshold	25% threshold
CHE incidence	1.72*** (0.47)	2.31*** (0.54)	2.01*** (0.59)
SHI	−0.56 (0.45)	−0.58 (0.45)	−0.59 (0.45)
CES-D score in baseline	0.25*** (0.02)	0.25*** (0.02)	0.25*** (0.02)
Controls	Yes	Yes	Yes
*N*	4,404	4,404	4,404
Subgroup analysis	Not receiving SHI	Receiving SHI	
CHE incidence (10% threshold)	3.08* (1.29)	1.50*** (0.35)	
Controls	Yes	Yes	
*N*	781	12,385	
Chow test	χ ^2^ (1) = 4.18, *p* = .041		

*Notes:* CHE = catastrophic health expenditure; CES-D = Center for Epidemiologic Studies—Depression; SHI = social health insurance. Clustered standard errors are presented in parentheses.

*p < .05, **p < .01, ***p < .001.

## Discussion and Conclusion

Our study presents two novel contributions to the existing mental health research. First, our analysis shows that incurring CHE is significantly associated with poor mental health, which is consistent with the broad body of literature that has found a direct impact of economic strains on mental health (Hiilamo & Grundy, 2020; Kourouklis et al., 2019; Li & Yuan, 2019; Wilkinson, 2016). Although there is no evidence that the receipt of SHI is directly associated with improved mental health, we found that the protective effect of SHI emerges once older people face CHE shocks. This is likely because SHI, like other social insurance schemes, reduces economic uncertainty or improves financial security (Sjoberg, 2010). For people experiencing CHE, insurance recipients are less worried about health expenditure getting out of control or incurring repeated CHE in the future in comparison to those not receiving SHI, so the worsening of mental health is lessened among insurance recipients. It is also worth noting that the association between CHE and mental health and the moderation effects of SHI remain significant after controlling for a variety of personal characteristics and individual-level RE/FE.

Second, the quantile regression analyses demonstrate that the relationships between mental health, CHE incidence, and SHI are heterogeneous for people with different levels of mental health problems. It seems that older people are most vulnerable to the negative impacts of CHE when they have mild depressive symptoms. For people with more severe depressive symptoms, the association between CHE and mental health becomes less pronounced. Meanwhile, the observed protective influences of SHI are also weaker. These results imply that, despite the crucial role of the household economy in older people’s mental health, simply alleviating the economic burden of health care or reducing economic uncertainty via social insurance may be insufficient for people living with severe depressive symptoms. Recovery from most severe depressive disorders requires high-quality mental health services and carefully designed intervention programs, both of which are lacking in the Chinese health care system.

Several policy implications can be drawn from our findings. First, the mental health aspect of CHE needs to be further addressed by the government. There is a clear association between incurring CHE and poor mental health, but in most LMICs, such as China, disease treatment is rarely integrated with psychologically informed approaches. Governments in LMICs should have a more systematic approach for experienced clinicians to work with patients and to address their psychological and emotional needs. There are some useful examples from which China can learn, such as Macmillan Cancer Support in the United Kingdom.

Second, the weakened association between CHE and mental health among people receiving SHI seems to provide a case for a more generous health financing mechanism. Our study is based on data from China, but our findings have important policy implications for other LMICs. SHI can be a starting point for governments in LMICs to consider this approach, which not only helps older people reduce the OOP burden but also has great potential in making a positive impact on people’s mental health.

Finally, generous financial support should be matched with focused attention on social equality. Our calculations suggest that a nontrivial proportion of the older population experienced CHE (i.e., 5.3%) if we set the catastrophic threshold at the 10% level. Health care expenditure is strongly driven by health care needs (You & Kobayashi, 2011). Like many other countries, poorer people in China have greater care needs than the rich and thus are more likely to face CHE (Yang & Kanavos, 2012). It is essential that the current SHI system provides sufficient benefits for the poorest segment of the older population and protects them from financial hardship caused by CHE.

The limitations of this study should be given due attention. First, although we controlled for important confounding factors and included the individual-level FEs in the analyses to address the potential endogeneity of the CHE variable, the possible reverse causality still limits our ability to interpret the results presented as causation. Second, CHE may have a delayed impact on mental health, which was not captured in our study. Finally, indirect health care costs such as transportation and accommodation were not included in the health care expenditure, so the incidence of CHE may have been underestimated.

## Supplementary Material

gbab130_suppl_Supplementary_Materials
