# Supplementary material for: Catastrophic Health Expenditure and Mental Health in the Older Chinese Population: The Moderating Role of Social Health Insurance
Source: J Gerontol B Psychol Sci Soc Sci. 2021 Jul 13;77(1):160–9. doi: 10.1093/geronb/gbab130 (PMC8755894; doi:10.1093/geronb/gbab130)
Supplement: gbab130_suppl_Supplementary_Materials [file gbab130_suppl_supplementary_materials.docx]

**Appendix**

Table A1 Association between CHE incidence, mental health, and SHI: panel data regression models

|  | **CHE (10%)** | | **CHE (20%)** | | **CHE (25%)** | |
| --- | --- | --- | --- | --- | --- | --- |
|  | RE | FE | RE | FE | RE | FE |
| Incidence of CHE | 3.68*** | 3.43* | 3.61*** | 4.01** | 2.74** | 2.95* |
|  | (0.98) | (1.44) | (1.09) | (1.31) | (1.03) | (1.28) |
| Having SHI or not | -0.14 | 0.12 | -0.17 | 0.09 | -0.20 | 0.04 |
|  | (0.24) | (0.37) | (0.24) | (0.37) | (0.24) | (0.37) |
| SHI × Incidence of CHE | -2.40* | -2.65 | -2.21* | -3.24* | -1.36 | -2.08 |
|  | (1.00) | (1.46) | (1.11) | (1.35) | (1.06) | (1.32) |
| Age | -0.03* | 0.02 | -0.03* | 0.02 | -0.03* | 0.03 |
|  | (0.01) | (0.09) | (0.01) | (0.09) | (0.01) | (0.09) |
| Female | 1.99*** | N.A. | 2.00*** | N.A. | 2.00*** | N.A. |
|  | (0.15) |  | (0.15) |  | (0.15) |  |
| Rural areas | 1.05*** | N.A. | 1.05*** | N.A. | 1.05*** | N.A. |
|  | (0.13) |  | (0.13) |  | (0.13) |  |
| Secondary education or below | -0.70*** | -0.88 | -0.70*** | -0.87 | -0.70*** | -0.88 |
|  | (0.14) | (1.00) | (0.14) | (1.00) | (0.14) | (1.00) |
| High school or above | -1.26*** | -2.40 | -1.25*** | -2.39 | -1.26*** | -2.41 |
|  | (0.22) | (1.46) | (0.22) | (1.46) | (0.22) | (1.46) |
| Married | 2.86*** | 2.95*** | 2.85*** | 2.93*** | 2.86*** | 2.94*** |
|  | (0.42) | (0.72) | (0.42) | (0.72) | (0.42) | (0.72) |
| Good health | 1.01*** | 0.33 | 1.01*** | 0.33 | 1.01*** | 0.33 |
|  | (0.14) | (0.21) | (0.14) | (0.21) | (0.14) | (0.21) |
| Fair or poor health | 2.12*** | 0.77*** | 2.13*** | 0.79*** | 2.14*** | 0.79*** |
|  | (0.15) | (0.22) | (0.15) | (0.22) | (0.15) | (0.22) |
| Number of chronic diseases | 0.57*** | 0.50*** | 0.58*** | 0.50*** | 0.58*** | 0.50*** |
|  | (0.05) | (0.13) | (0.05) | (0.13) | (0.05) | (0.13) |
| Number of ADL limitations | -0.04 | 0.42* | -0.04 | 0.43* | -0.04 | 0.42* |
|  | (0.17) | (0.21) | (0.17) | (0.21) | (0.17) | (0.21) |
| Number of IADL limitations | 0.50*** | 0.42** | 0.50*** | 0.41** | 0.50*** | 0.42** |
|  | (0.10) | (0.14) | (0.10) | (0.14) | (0.10) | (0.14) |
| Having pain | 3.01*** | 1.78*** | 3.01*** | 1.78*** | 3.01*** | 1.78*** |
|  | (0.13) | (0.18) | (0.13) | (0.18) | (0.13) | (0.18) |
| Smoke | 1.22*** | 0.56* | 1.21*** | 0.56* | 1.21*** | 0.56* |
|  | (0.14) | (0.24) | (0.14) | (0.24) | (0.14) | (0.24) |
| Drinking | 0.03 | 0.5 | 0.03 | 0.51 | 0.03 | 0.51 |
|  | (0.14) | (0.27) | (0.14) | (0.27) | (0.14) | (0.27) |
| Household expenditure | -0.14* | 0.01 | -0.14* | 0.01 | -0.14* | 0.01 |
|  | (0.06) | (0.10) | (0.06) | (0.10) | (0.06) | (0.10) |
| Total health expenditure | -0.02 | -0.02 | -0.02 | -0.02 | -0.02 | -0.02 |
|  | (0.01) | (0.01) | (0.01) | (0.01) | (0.01) | (0.01) |
| Year=2013 | 0.23 | -0.27 | 0.24 | -0.26 | 0.24 | -0.27 |
|  | (0.13) | (0.23) | (0.13) | (0.24) | (0.13) | (0.24) |
| Year=2015 | 0.77*** | 0.34 | 0.78*** | 0.36 | 0.79*** | 0.33 |
|  | (0.13) | (0.38) | (0.13) | (0.38) | (0.13) | (0.38) |

Notes: clustered standard errors are presented in the parentheses; * *p*<0.05, ** *p*<0.01, *** *p*<0.001; total health expenditure includes out-of-pocket health expenditure and expenditure paid by the government; CHE: catastrophic healthcare expenditure; SHI: social health insurance; RE: random effects model; FE: fixed effects model; N=13,166.

Table A2 Association between OOP health expenditure intensity, mental health, and SHI: panel data regression models

|  | Random effects model | Fixed effects model |
| --- | --- | --- |
| OOP health expenditure | 0.41** | 0.51*** |
|  | (0.16) | (0.15) |
| Having SHI or not | -0.18 | 0.04 |
|  | (0.24) | (0.37) |
| SHI × OOP health expenditure | -0.19 | -0.49 |
|  | (0.25) | (0.31) |
| Age | -0.03** | 0.03 |
|  | (0.01) | (0.09) |
| Female | 1.57*** | N.A. |
|  | (0.13) |  |
| Rural areas | 1.07*** | N.A. |
|  | (0.13) |  |
| Secondary education or below | -0.73*** | -0.93 |
|  | (0.14) | (1.01) |
| High school or above | -1.31*** | -2.4 |
|  | (0.22) | (1.47) |
| Married | 2.94*** | 2.97*** |
|  | (0.42) | (0.73) |
| Good health | 1.01*** | 0.32 |
|  | (0.14) | (0.21) |
| Fair or poor health | 2.16*** | 0.78*** |
|  | (0.15) | (0.22) |
| Number of chronic diseases | 0.57*** | 0.50*** |
|  | (0.05) | (0.13) |
| Number of ADL limitations | -0.07 | 0.42* |
|  | (0.17) | (0.21) |
| Number of IADL limitations | 0.50*** | 0.41** |
|  | (0.10) | (0.14) |
| Having pain | 3.03*** | 1.78*** |
|  | (0.13) | (0.18) |
| Household expenditure | -0.15* | 0.00 |
|  | (0.06) | (0.10) |
| Year=2013 | 0.04 | -0.37 |
|  | (0.12) | (0.23) |
| Year=2015 | 0.77*** | 0.31 |
|  | (0.13) | (0.38) |

Notes: clustered standard errors are presented in the parentheses; * *p*<0.05, ** *p*<0.01, *** *p*<0.001; CHE: catastrophic healthcare expenditure; SHI: social health insurance; N=13,166.

Table A3. Association between CHE incidence and mental health: controlling for mental health in the baseline survey

|  | 10% threshold | | 20% threshold | 25% threshold |
| --- | --- | --- | --- | --- |
| CES-D score in baseline | 0.25*** | | 0.25*** | 0.25*** |
|  | (0.02) | | (0.02) | (0.02) |
| Incidence of CHE | 1.72*** | | 2.31*** | 2.01*** |
|  | (0.47) | | (0.54) | (0.59) |
| Having SHI or not | -0.56 | | -0.58 | -0.59 |
|  | (0.45) | | (0.45) | (0.45) |
| Age | -0.05** | | -0.05** | -0.05** |
|  | (0.02) | | (0.02) | (0.02) |
| Female | 1.01*** | | 1.02*** | 1.00*** |
|  | (0.22) | | (0.22) | (0.22) |
| Rural areas | 0.60** | | 0.62** | 0.62** |
|  | (0.20) | | (0.20) | (0.20) |
| Secondary education or below | -0.80*** | | -0.80*** | -0.80*** |
|  | (0.20) | | (0.20) | (0.20) |
| High school or above | -1.07** | | -1.09** | -1.09** |
|  | (0.36) | | (0.37) | (0.37) |
| Married | -0.44 | | -0.47 | -0.45 |
|  | (0.55) | | (0.55) | (0.55) |
| Good health | 0.86*** | | 0.85*** | 0.85*** |
|  | (0.22) | | (0.22) | (0.22) |
| Fair or poor health | 1.71*** | | 1.72*** | 1.74*** |
|  | (0.23) | | (0.23) | (0.23) |
| Number of chronic diseases | 0.46*** | | 0.47*** | 0.47*** |
|  | (0.07) | | (0.07) | (0.07) |
| Number of ADL limitations | 0.50* | | 0.49* | 0.50* |
|  | (0.22) | | (0.22) | (0.22) |
| Number of IADL limitations | 0.51*** | | 0.51*** | 0.51*** |
|  | (0.15) | | (0.15) | (0.15) |
| Having pain | 2.95*** | | 2.94*** | 2.95*** |
|  | (0.20) | | (0.20) | (0.20) |
| Smoke | 0.59** | | 0.58* | 0.56* |
|  | (0.23) | | (0.23) | (0.23) |
| Drinking | -0.07 | | -0.07 | -0.08 |
|  | (0.20) | | (0.20) | (0.20) |
| Household expenditure | -0.04 | | -0.03 | -0.03 |
|  | (0.10) | | (0.10) | (0.10) |
| Total health expenditure | -0.03 | | -0.04* | -0.04* |
|  | (0.02) | | (0.02) | (0.02) |
| Year=2015 | | 0.74*** | 0.73*** | 0.74*** |
|  | (0.16) | | (0.16) | (0.16) |
| R-square | 0.32 | | 0.32 | 0.32 |

Notes: clustered standard errors are presented in the parentheses; * *p*<0.05, ** *p*<0.01, *** *p*<0.001; CHE: catastrophic healthcare expenditure; SHI: social health insurance; N=4,404.

Table A4. Association between CHE incidence and mental health: subgroup analysis

|  | Insured by SHI | | Chronic diseases | |
| --- | --- | --- | --- | --- |
|  | No | Yes | One | Two or more |
| CHE (10% threshold) | 3.08* | 1.50*** | 2.59*** | 1.12* |
|  | (1.29) | (0.35) | (0.56) | (0.46) |
| SHI | N.A. | N.A. | -1.07* | -0.44 |
|  |  |  | (0.43) | (0.44) |
| Age | -0.09* | -0.02* | -0.03 | -0.02 |
|  | (0.04) | (0.01) | (0.02) | (0.02) |
| Female | 0.64 | 1.95*** | 1.62*** | 2.42*** |
|  | (0.60) | (0.16) | (0.27) | (0.24) |
| Rural areas | 1.59** | 0.93*** | 0.79*** | 1.25*** |
|  | (0.55) | (0.13) | (0.24) | (0.21) |
| Secondary education or below | -0.62 | -0.72*** | -0.41 | -1.13*** |
|  | (0.53) | (0.14) | (0.24) | (0.23) |
| High school or above | -1.46 | -1.17*** | -0.84* | -1.67*** |
|  | (0.76) | (0.23) | (0.41) | (0.37) |
| Married | 4.43** | 2.69*** | 2.99*** | 2.17** |
|  | (1.68) | (0.42) | (0.76) | (0.68) |
| Good health | 1.40* | 1.18*** | 1.05*** | 1.51*** |
|  | (0.63) | (0.15) | (0.26) | (0.34) |
| Fair or poor health | 1.95** | 2.57*** | 2.33*** | 3.14*** |
|  | (0.67) | (0.15) | (0.26) | (0.33) |
| Number of chronic diseases | 0.49** | 0.54*** | N.A. | 0.61*** |
|  | (0.50) | (0.19) |  | (0.23) |
| Number of ADL limitations | 0.21 | -0.06 | 0.26 | -0.05 |
|  | (0.50) | (0.19) | (0.33) | (0.23) |
| Number of IADL limitations | 0.6 | 0.55*** | 0.56** | 0.38** |
|  | (0.43) | (0.11) | (0.20) | (0.14) |
| Having pain | 3.64*** | 3.49*** | 3.80*** | 3.37*** |
|  | (0.59) | (0.14) | (0.25) | (0.20) |
| Smoke | -0.34 | 1.39*** | 1.18*** | 1.77*** |
|  | (0.61) | (0.15) | (0.26) | (0.25) |
| Drinking | -0.76 | -0.01 | -0.50* | 0.08 |
|  | (0.61) | (0.14) | (0.24) | (0.23) |
| Household expenditure per capita | -0.56* | -0.15* | -0.35** | -0.11 |
|  | (0.28) | (0.07) | (0.11) | (0.11) |
| Total health expenditure | 0.01 | -0.03* | -0.02 | -0.02 |
|  | (0.04) | (0.01) | (0.02) | (0.02) |
| Year=2013 | 0.05 | 0.42** | 0.27 | 0.38 |
|  | (0.71) | (0.14) | (0.24) | (0.21) |
| Year=2015 | 0.68 | 0.87*** | 0.56* | 1.05*** |
|  | (0.59) | (0.14) | (0.24) | (0.22) |
| N | 781 | 12,385 | 3,801 | 5,441 |
| R-square | 0.22 | 0.20 | 0.18 | 0.18 |

Notes: clustered standard errors are presented in the parentheses; * *p*<0.05, ** *p*<0.01, *** *p*<0.001; CHE: catastrophic healthcare expenditure; SHI: social health insurance.

Table A5. Sensitivity analysis: controlling for household income

|  | **Panel data regression** | | **Quantile regression** | | | | |
| --- | --- | --- | --- | --- | --- | --- | --- |
|  | **Random effects** | **Fixed effects** | **0.1** | **0.3** | **0.5** | **0.7** | **0.9** |
| CHE (10% threshold) | 3.70*** | 3.43* | 4.40*** | 3.00* | 4.36* | 3.83** | 1.44* |
|  | (0.99) | (1.44) | (1.28) | (1.17) | (2.00) | (1.36) | (0.72) |
| SHI | -0.11 | 0.12 | 0.12 | -0.05 | -0.14 | -0.71* | -1.00* |
|  | (0.24) | (0.37) | (0.10) | (0.25) | (0.31) | (0.33) | (0.51) |
| SHI × Incidence of CHE | -2.41* | -2.65 | -3.74** | -1.96 | -3.02 | -2.23 | 1.50* |
|  | (1.01) | (1.46) | (1.33) | (1.19) | (2.00) | (1.32) | (0.72) |
| Age | -0.03* | 0.02 | -0.01* | -0.02* | -0.02* | -0.02 | -0.02 |
|  | (0.01) | (0.09) | (0.01) | (0.01) | (0.01) | (0.01) | (0.01) |
| Female | 1.98*** | N.A. | 0.78*** | 1.19*** | 1.54*** | 2.11*** | 2.30*** |
|  | (0.15) |  | (0.11) | (0.12) | (0.14) | (0.17) | (0.21) |
| Rural areas | 0.97*** | N.A. | 0.49*** | 0.90*** | 0.80*** | 0.74*** | 1.17*** |
|  | (0.14) |  | (0.07) | (0.11) | (0.12) | (0.15) | (0.19) |
| Secondary education or below | -0.67*** | -0.88 | -0.30*** | -0.35** | -0.65*** | -1.03*** | -0.93*** |
|  | (0.14) | (1.00) | (0.06) | (0.12) | (0.12) | (0.16) | (0.19) |
| High school or above | -1.13*** | -2.40 | -0.23* | -0.57*** | -1.15*** | -1.32*** | -1.42** |
|  | (0.23) | (1.46) | (0.11) | (0.16) | (0.22) | (0.26) | (0.44) |
| Married | 2.79*** | 2.95*** | 9.99*** | 2.21*** | 1.00* | 0.5 | 0.09 |
|  | (0.42) | (0.72) | (0.09) | (0.48) | (0.45) | (0.51) | (0.18) |
| Good health | 0.98*** | 0.33 | 0.44*** | 0.81*** | 1.05*** | 1.24*** | 2.09*** |
|  | (0.14) | (0.21) | (0.05) | (0.12) | (0.14) | (0.19) | (0.24) |
| Fair or poor health | 2.09*** | 0.78*** | 0.89*** | 1.70*** | 2.35*** | 2.86*** | 4.24*** |
|  | (0.15) | (0.22) | (0.10) | (0.13) | (0.15) | (0.18) | (0.25) |
| Number of chronic diseases | 0.57*** | 0.50*** | 0.34*** | 0.51*** | 0.57*** | 0.58*** | 0.48*** |
|  | (0.05) | (0.13) | (0.03) | (0.05) | (0.05) | (0.05) | (0.06) |
| Number of ADL limitations | -0.05 | 0.42* | -1.59*** | 0.23 | 0.45** | 0.57** | 0.41** |
|  | (0.17) | (0.21) | (0.15) | (0.23) | (0.16) | (0.18) | (0.13) |
| Number of IADL limitations | 0.50*** | 0.42** | -0.31* | 0.62*** | 0.94*** | 1.01*** | 0.98*** |
|  | (0.10) | (0.14) | (0.14) | (0.12) | (0.10) | (0.13) | (0.08) |
| Having pain | 2.98*** | 1.78*** | 1.64*** | 2.80*** | 3.61*** | 4.32*** | 4.87*** |
|  | (0.13) | (0.18) | (0.16) | (0.15) | (0.14) | (0.18) | (0.19) |
| Smoke | 1.21*** | 0.56* | 0.65*** | 0.73*** | 0.79*** | 0.97*** | 0.96*** |
|  | (0.14) | (0.24) | (0.08) | (0.13) | (0.13) | (0.18) | (0.23) |
| Drinking | 0.02 | 0.5 | 0.09 | 0.11 | -0.06 | -0.07 | -0.22 |
|  | (0.14) | (0.27) | (0.06) | (0.11) | (0.12) | (0.16) | (0.20) |
| Household income | -1.58*** | 0.19 | -0.84*** | -1.44*** | -2.35*** | -2.58*** | -3.39*** |
|  | (0.45) | (0.66) | (0.20) | (0.27) | (0.39) | (0.39) | (0.43) |
| Total health expenditure | -0.02 | -0.02 | -0.02 | -0.01 | -0.02 | -0.03** | -0.04*** |
|  | (0.01) | (0.01) | (0.02) | (0.01) | (0.02) | (0.01) | (0.01) |
| Year=2013 | 0.20 | -0.27 | 0.86*** | 0.26 | -0.21 | -0.56** | -1.05*** |
|  | (0.13) | (0.23) | (0.09) | (0.13) | (0.14) | (0.17) | (0.22) |
| Year=2015 | 0.68*** | 0.35 | 0.71*** | 0.11 | -0.1 | -0.04 | -0.06 |
|  | (0.12) | (0.38) | (0.06) | (0.12) | (0.14) | (0.17) | (0.20) |

Notes: cluster/heteroskedasticity-robust standard errors are presented in the parentheses; * *p*<0.05, ** *p*<0.01, *** *p*<0.001; total health expenditure includes out-of-pocket health expenditure and expenditure paid by the government; CHE: catastrophic healthcare expenditure; SHI: social health insurance; N=13,166.
